# Supplementary figures and images for: CellTracksColab is a platform that enables compilation, analysis, and exploration of cell tracking data
Source: PLoS Biol. 2024 Aug 8;22(8):e3002740. doi: 10.1371/journal.pbio.3002740 (PMC11335138; doi:10.1371/journal.pbio.3002740)

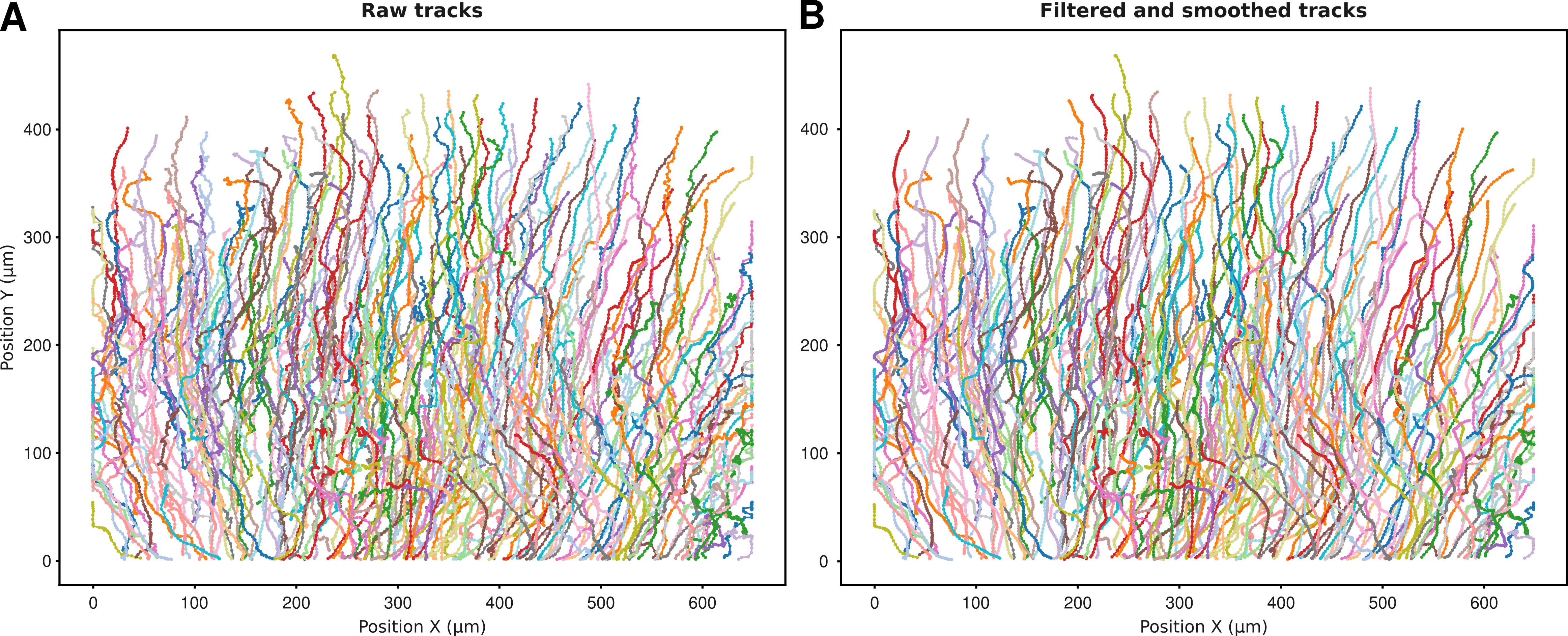

Supplement: S1 Fig — Example of track display before (A) and after (B) smoothing and filtering. The tracks originate from a video of migrating breast cancer cells tracked from their nuclei using TrackMate. (TIF) [file pbio.3002740.s001.tif]

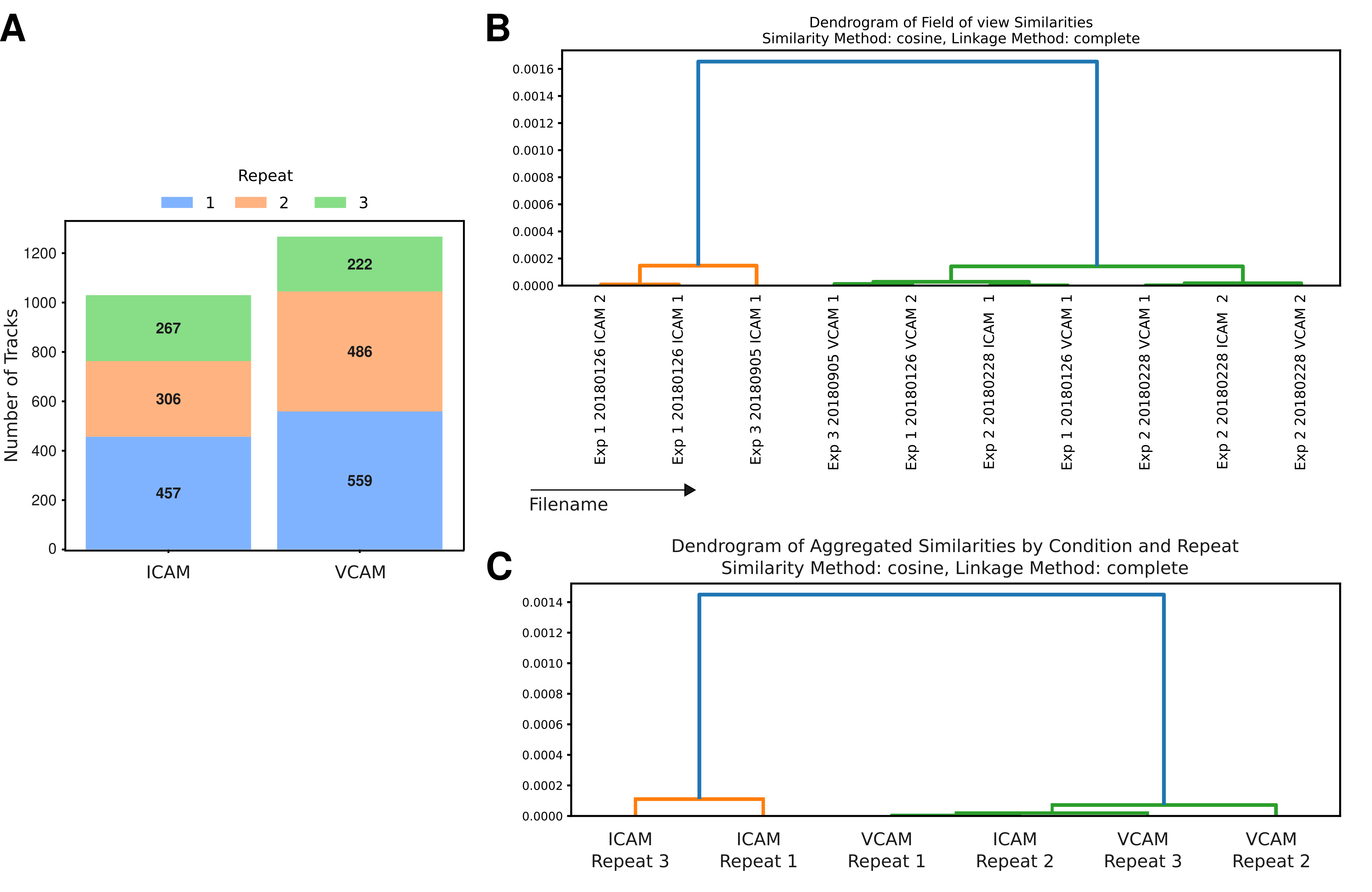

Supplement: S2 Fig — (A) This panel presents a stacked histogram showcasing the number of tracks for each biological repeat under different conditions, aiding in evaluating the dataset’s balance. Each biological repeat is color-coded, and each histogram segment’s specific number of tracks is annotated. (B, C) Hierarchical clustering: These dendrograms reveal the hierarchical clustering within the dataset by utilizing the cosine similarity metric and a complete linkage method. (B) FOV-based clustering analysis: This dendrogram illustrates the clustering across the 10 available fields of view (FOVs). (C) Condition and repeat-based clustering: This dendrogram delves deeper by segregating the dataset based on conditions and biological repeats. The dataset, including the raw images, the tracking files, and all the CellTracksColab results (including numerical data), are also available on Zenodo (11282716). (TIF) [file pbio.3002740.s002.tif]

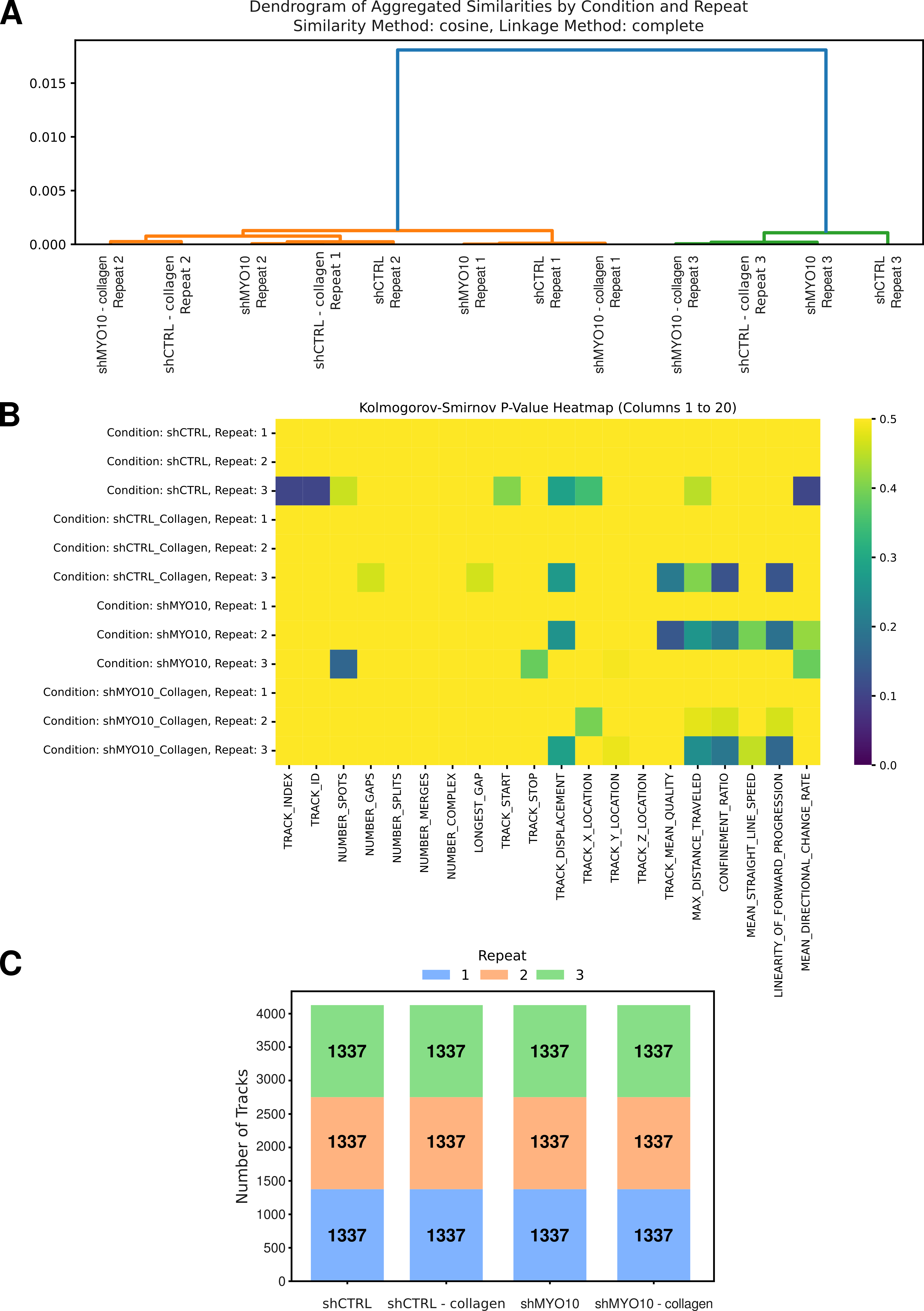

Supplement: S3 Fig — (A) This dendrogram utilizes the cosine similarity metric and a complete linkage method to assess the similarity in the dataset between conditions and biological repeats. (B) p-Value heatmap comparing the differences between the data distribution before and after resampling for each condition and repeats (selected number of track metrics). (C) This panel presents a stacked histogram showcasing the number of tracks for each biological repeat under different conditions, aiding in evaluating the dataset’s balance. Each biological repeat is color-coded, and each histogram segment’s specific number of tracks is annotated. The dataset, including the raw images, the tracking files, and all the CellTracksColab results (including numerical data), are also available on Zenodo (11282716). (TIF) [file pbio.3002740.s003.tif]

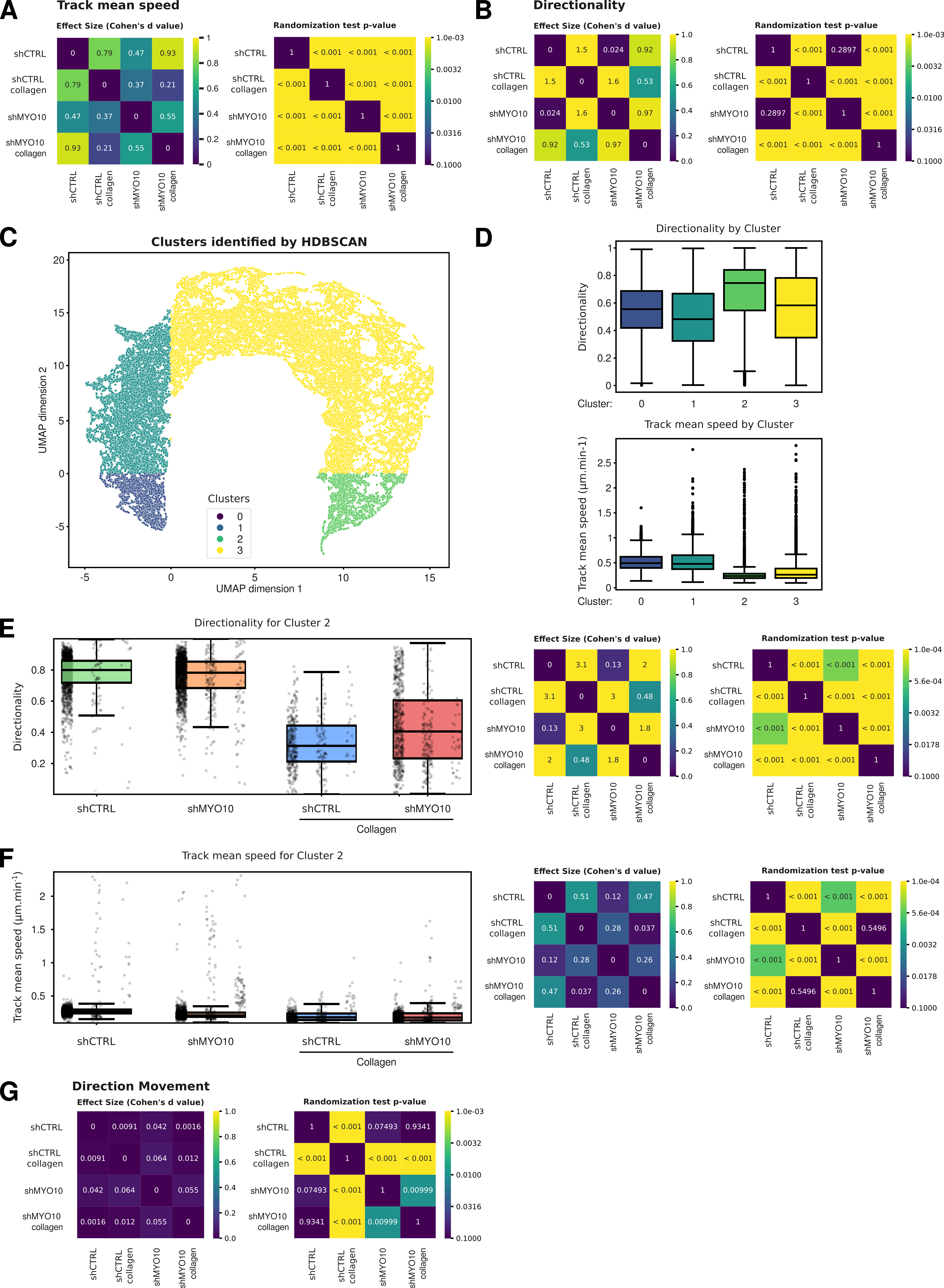

Supplement: S4 Fig — (A, B) p-Value and Cohen’s d value mirrored heatmaps for the “track mean speed” (A) and track “directionality” (B) metrics (see Fig 3C). (C) 2D UMAP projection of the entire breast cancer migration dataset, using all available track metrics for dimensionality reduction. Resultant clusters from the HDBSCAN analysis on the 2D UMAP projection. The Canberra method served as the metric for clustering. Each identified cluster is color-coded. (D) The “track mean speed” and track “directionality” for each cluster are summarized in a Tukey boxplot format. (E, F) The track “directionality” (E) and “track mean speed” (F) metrics for each condition for cluster 2 are summarized in a Tukey boxplot format. For all box plots, the vertical whiskers extend to data points within 1.5× the interquartile range, and the values for each track are shown as dots where each biological replicate is displayed next to each other from R1 to R3 (left to right). p-Value and Cohen’s d value mirrored heatmaps are displayed on the right. (G) p-Value and Cohen’s d value mirrored heatmaps for the “Direction Movement” metric (see Fig 3G). (D, E, and F) Underlying numerical data can be found in S1 Data. The dataset, including the raw images, the tracking files, and all the CellTracksColab results (including numerical data), are also available on Zenodo (11282716). (TIF) [file pbio.3002740.s004.tif]

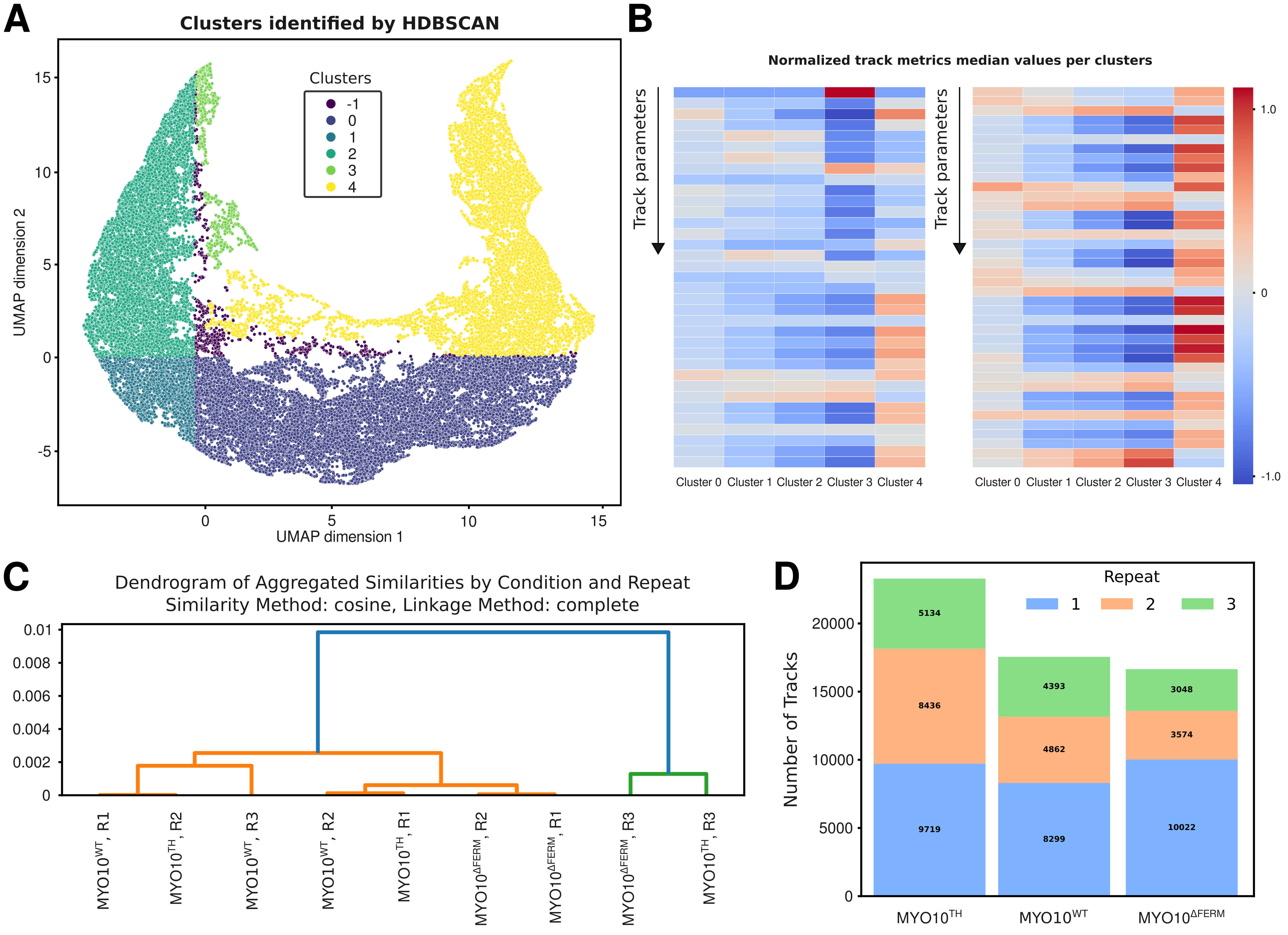

Supplement: S5 Fig — (A) 2D UMAP projection of the entire filopodia dataset, using all available track metrics for dimensionality reduction. Resultant clusters from the HDBSCAN analysis on the 2D UMAP projection. The Canberra method served as the metric for clustering. Each identified cluster is color-coded. (B) Heatmap representation, normalized using Z-scores, displaying variations in selected track metrics among the clusters. Full heatmaps are available in the Zenodo archive of this dataset. (C) This dendrogram utilizes the cosine similarity metric and a complete linkage method to assess the similarity in the filopodia dataset between conditions and biological repeats. (D) This panel presents a stacked histogram showcasing the number of tracks for each biological repeat under different conditions. Each biological repeat is color-coded, and each histogram segment’s specific number of tracks is annotated. The dataset, including the raw images, the tracking files, and all the CellTracksColab results (including numerical data), are also available on Zenodo (11285514). (TIF) [file pbio.3002740.s005.tif]
