# Supplementary material for: CellTracksColab is a platform that enables compilation, analysis, and exploration of cell tracking data
Source: PLoS Biol. 2024 Aug 8;22(8):e3002740. doi: 10.1371/journal.pbio.3002740 (PMC11335138; doi:10.1371/journal.pbio.3002740)
Supplement: Attachment — Submitted filename: Response to Reviewers.pdf [file pbio.3002740.s008.pdf]

Reviewer #1: Jacquemet presents a tool - CellTracksColab - that relies on Google resources to analyze biological tracking data. I like the idea behind the platform: a simple way to give non-programmers access to advanced analysis. Personally, I would not use it nor encourage my lab to do so (we would need more flexibility and the Google tie-in is a killer), but I can see the utility of the platform. From a publication point of view, there is not so much innovation in the analysis, but that isn't the point. The point is to bring currently available tools to the masses and the platform can definitely meet this goal. I like the way the platform is presented, allowing users to go deeper with successive routines if they want. I don't imaging many novices will want a t-SNE plot of their tracks, but it's there if they do.

We thank the reviewer for taking the time to assess our work and to provide valuable feedback. We appreciate your detailed review and acknowledge your point regarding the platform's flexibility and dependency on Google resources. We have enhanced the platform's adaptability, including enabling local usage via Jupyter Notebooks (https://github.com/CellMigrationLab/CellTracksColab/wiki/Running-CellTracksColab-locally) and improving support for TrackMate XML format (https://github.com/CellMigrationLab/CellTracksColab/wiki/The-TrackMate-notebook#trackmate-xml-files) and plotting functionalities. These updates address the critical issues raised, and we hope our revised manuscript meets your approval for publication. Thank you again for your valuable feedback.

The USP is using Google resources, but this is a serious limitation in my view: a) the user needs sufficient privileges to do their analyses (the free tier is restricted), b) there is an issue over data privacy, and even needing a Google account is a stumbling block for anyone concerned about their privacy, c) Google is notorious for shuttering useful platforms - this could all disappear tomorrow, d) uploading massive datasets to do analysis is an accessibility issue. To be fair, the author acknowledges a and b in the manuscript, and offers "To address this challenge, we plan to enable CellTracksColab notebooks to function seamlessly across different platforms via automatically generated Docker images". To my mind, this step is required for publication. This shouldn't all hang off Google and the ability to run this locally, means points a to d are nullified. I would not advocate a Docker image as there are a separate set of issues with that, but it would be straightforward for the author to generate a conda/yml install for people to just run all this stuff in a Jupyter notebook on their machine. If they did this, it would increase the use of the platform. I would be happy to use a local version, but not the online one. Speaking from the experience of using ColabFold, for lightweight predictions performed occasionally, it's OK; for anything even vaguely serious, you need a local install of AlphaFold. While that requires a lot of computing resources, what is being used here is a simple means to run a bit of python code (there isn't a strong case to use Google computing resources for these analyses) locally, and I hope the author agrees this is worth doing.

We understand the concerns raised by the reviewer about the reliance on Google Colab and its limitations, especially in terms of data privacy, the necessity of a Google account, and the potential discontinuation of the platform. In response, we have enhanced the versatility of CellTracksColab by providing comprehensive instructions for running the platform locally using Jupyter Notebooks, now available on our GitHub page (including video protocols). While, Jupyter Notebooks do not currently support code hiding natively; we have encapsulated the visible code within external functions, significantly improving the clarity of the user interface and reducing potential intimidation for non-programmers, making the platform more user-friendly for all.

https://github.com/CellMigrationLab/CellTracksColab/wiki/Running-CellTracksColab-locally

As most of the code is now externally packaged, the functionality can also be called outside a notebook environment.

For users who prefer Google Colab's interface but are concerned about the limitations mentioned, we offer a hybrid solution where Google Colab can run in a local environment. This approach leverages Colab's user-friendly interface while running computations on the user's local resources, thus mitigating data privacy concerns and the dependency on Google's cloud services. We also provide detailed documentation on how to use this third option.

https://github.com/CellMigrationLab/CellTracksColab/wiki/Running-CellTracksColab-locally#running-celltrackscolab-notebooks-in-jupyter-lab

We believe these enhancements significantly improve the accessibility and user experience of CellTracksColab, broadening its applicability and ensuring it remains a valuable tool for the scientific community.

The second major limitation is that while it is billed as a "do everything" platform, it only takes TrackMate output. If the tracking is done using other software, the outputs would require transforming into TrackMate format. This isn't so clear from my reading of the paper.

Thank you for highlighting this limitation. We acknowledge the need for a more precise explanation regarding the data formats supported by CellTracksColab. Initially, our platform was tested to work primarily with CSV files generated by TrackMate and custom CSV files such as those created by the Manual Tracker in Fiji. Recognizing the diversity of tracking software used in the field, we have now tested our platform's compatibility with various popular open-source tracking software such as Trackmate, CellProfiler, Icy, ilastik, and the Fiji Manual Tracker, as specified in our revised manuscript:

“CellTracksColab is designed to process tracking data from various popular open-source tracking software, including Trackmate[1], CellProfiler[12], Icy[13], ilastik[14], and the Fiji Manual Tracker[15]. CellTracksColab supports tracking data stored in XML

(TrackMate) and CSV formats (TrackMate, CellProfiler, Icy, ilastik, and Fiji Manual Tracker). CellTracksColab may also be compatible with other tracking software that exports results compatible with our minimal requirements (see documentation for details).”

To assist users in integrating data from various sources, we have updated our documentation on GitHub:

https://github.com/CellMigrationLab/CellTracksColab/wiki/Data-requirements-and-supported-software

We also commit to including other options in the future, depending on our users' requests.

The other limitation is the use of 3 csv files per tracked experiment. It would be more efficient for users to save the TrackMate XML file and for CellTracksColab to parse that, rather than deal with multiple files. There would be less user error here too. Does the platform currently check that all files are present? Finally, it's on the user to ensure the scaling information of their tracking data is correct. Users, particularly novices, tend to lose the scaling information when they process images. It would be useful if there could be a sanity check implemented. A simple check is if the spatial units are pixels, then warn the user that their results are not scaled. This is harder to do from the CSVs
and would be trivial if the platform used TrackMate XML instead. If the author could acknowledge and/or address these limitations, it would improve the platform and manuscript.

Thank you for your insightful comments. We value your feedback and it plays a crucial role in our software's development. We initially opted for CSV files due to their smaller size, which simplifies the uploading process to our online platform and minimizes upload costs for users. Recognizing the limitations you pointed out, we have now implemented an XML loader that allows for direct reading and importation of TrackMate XML files into CellTracksColab. This enhancement is particularly beneficial for local use, where file size is less of a concern. Additionally, during the import of TrackMate CSV or XML files, the platform now prints a message indicating the pixel calibration settings. We have also introduced checks to ensure all necessary files are present, minimizing the potential for user error.

Very specific points:
1. The heatmap for Cohen's d which is [0,1] works well. The heatmap for p-values, not so much. First, the colorscale needs to be log transformed for this to be useful. It is also not possible to have p-values that equal 0 or 1, this simply means the numeric precision of the labels used as overlays in the heatmap are insufficient. What would work better here is to not show labels that are ~ 1 (or > 0.1 or some other cut off), then show the -log10 transform of the p-value. So that 1 x 10^-6 can be discerned from 1 x 10^-5.

Thank you for your feedback on the visualization of p-values in our heatmaps. We have considered your suggestions and made significant improvements to the display. The color scale for the p-values is now log-transformed, enhancing the differentiation and readability of significant versus non-significant values. Additionally, we have adjusted the numeric precision of the p-value labels to avoid displaying values as 0 or 1, which can be misleading.

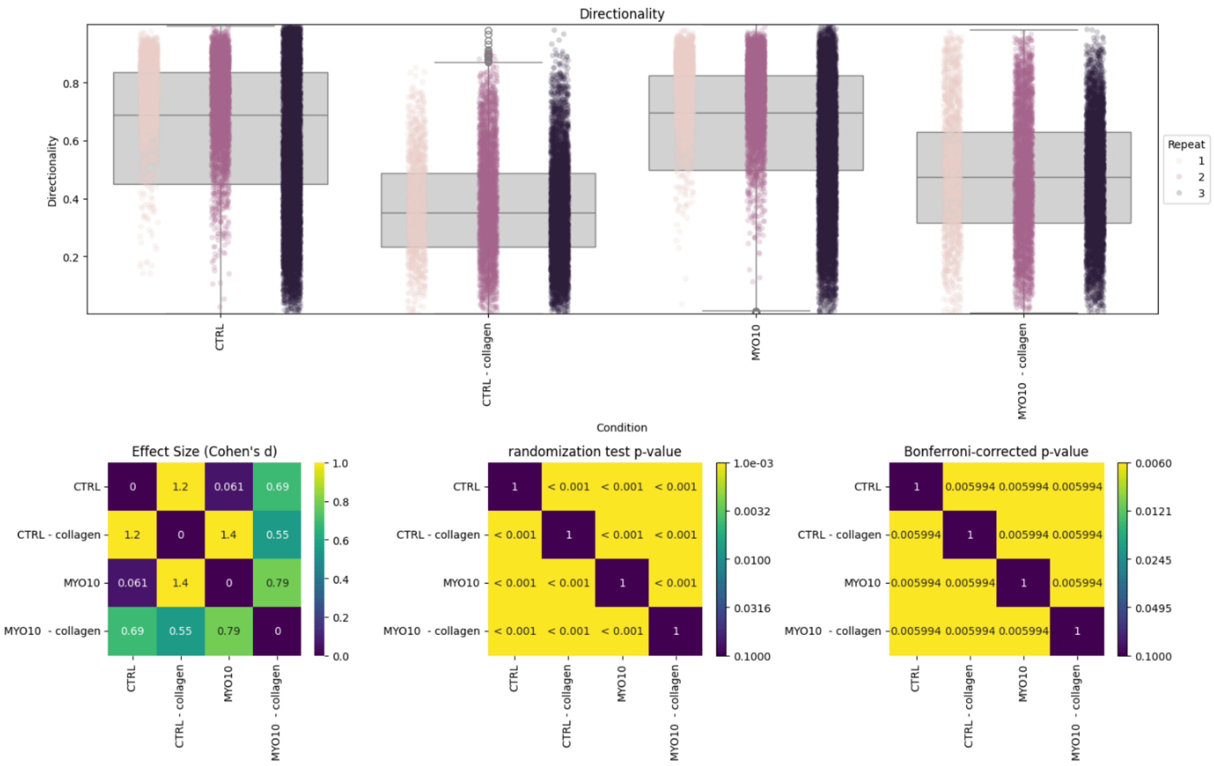

2. As a follow up, are those p-values shown in Fig 1C calculated correctly? As in, using the means of the repeats (n = 3 for each, superplot style) or using all the tracks (n > 1000s, pseudoreplication)

Thank you for your question about the calculation of p-values. In our initial submission, these values were derived from a randomization test combined with Cohen's d value. This method is particularly effective because it leverages the robustness of non-parametric testing, which does not assume a normal distribution of the data and is less sensitive to outliers, providing a more reliable assessment under various data distributions. This strategy is also advantageous when the number of biological repeats is low.

In response to your excellent suggestion, we have expanded our analysis options to include t-tests that calculate p-values based on the means of the repeats, as described in the SuperPlots methodology. This addition enhances the platform's flexibility, allowing users to

choose between detailed individual track analyses using the entire dataset or aggregated data analyses that focus on high-level trends based on the means of biological repeats.

https://github.com/CellMigrationLab/CellTracksColab/wiki/Plotting-Track-Metrics#statistical-analyses

3. the link on GitHub "Data Structure: Organize with our two-tiered folder hierarchy" is currently broken

Thank you, the link is now fixed.

4. The implementation of Ripley's L function shown in Figure 1, how does it deal with search areas that go beyond the image? For low density images like the one shown in 1F this is a significant issue with the calculation.

We thank the reviewer for raising this critical point. In the image shown in Figure 1F, only a single point is depicted, not all the points from the image. However, the reviewer's concern about the calculation for other scenarios is valid. In our current implementation of Ripley's L function, we address edge effects using the following approach:

1. Before calculating, we ensure that the maximum search radius does not exceed the image's dimensions. If it is larger, the system displays an error message to prevent the calculation from proceeding.
2. We identify points that are closer to the edges than the maximum search radius. The code informs users about the number of these edge points to maintain awareness of potential edge-related biases. Affected points are labeled in a different color to facilitate easy visualization. This helps users identify and consider these points separately if necessary.
3. During significance testing (Monte Carlo simulations), random points are generated within the full image dimensions. This approach ensures that edge effects are consistently considered across different datasets.

By implementing these steps, we hope to minimize edge effects and provide accurate spatial analysis results.

5. Kudos to the author for making some test datasets available. My point above about the XML files, means that the tracking can be reproduced because the parameters for TrackMate are stored in the XML file, which is not the case for csv. You can tell I am not a fan of the csv files!

Thank you for acknowledging our efforts to make test datasets available, which are crucial for validating and reproducing research findings. We appreciate your feedback regarding

the format of these datasets. In response to your comments, we have now enabled CellTracksColab to support XML file ingestion. This allows for the integration of TrackMate data, ensuring that all parameters involved in the tracking process are retained and can be fully reproduced. This addresses one of the main concerns associated with CSV files. However, we continue to support CSV formats due to their accessibility and ease of user manipulation. CSV files are widely used because they are readable and editable with simple text editors or spreadsheet software, making them a practical option for users who wish to manually review or adjust data. Offering both XML and CSV compatibility allows users to choose the format that best suits their needs, enhancing the usability and flexibility of CellTracksColab.

6. The author makes the case for analysis of all kinds of tracking data from molecules to whole animals. Have they tested whether CellTracksColab can handle all different kinds of data? Maybe if users report problems tracking objects different to cancer cells or filopodia, they can be patched on-the-fly? It would be good to know if there has been some stress testing done, because the initial parameters offered by CellTracksColab are unlikely to work for every case.

Thank you for your inquiry regarding the versatility of CellTracksColab and its capacity to handle diverse types of tracking data. Our development efforts have primarily centered on applications within cell biology, where we have conducted extensive testing with various types of cell-tracking datasets, such as those involving cancer cells and filopodia. As outlined in the manuscript, it's important to note that CellTracksColab is not currently optimized for lineage tracing analyses, which may limit its use in certain specialized biological studies. Despite these limitations, CellTracksColab is fundamentally designed to process and analyze tracking data based on x, y, and z coordinates, which suggests its potential applicability across a wide range of scientific domains. This could include tracking studies ranging from molecular dynamics to tracking whole animals in ecological or behavioral contexts.

Reviewer #2: Please see attachment, which includes the review along with figures highlighting some minor issues with the code. The authors of CellTracksColab present an interesting platform for analysing image tracking data, which appears very useful for the community. However, some points should be addressed to make the suggested tool more accessible.

We thank the reviewer for taking the time to evaluate our manuscript and to provide feedback on CellTracksColab.

One question is regarding how some of their metrics are calculated within the platform and which parameters can be tuned. For example, the smoothing option is described in the

notebook as "Smoothing: Adjust this slider to control the degree of smoothing you'd like to apply to your tracks." - what is the slider defining? The temporal interval of a moving average? It is possible to retrieve this information by expanding the code cells in the notebook, but this is only true for experienced (coding) users.

Thank you for bringing this point up. The "Smoothing" slider in the platform controls the temporal interval of a moving average applied to the tracking data. This means that the slider adjusts how many consecutive frames are considered for averaging to smooth the trajectory data. Recognizing the need for clarity, we have significantly improved the platform documentation associated with the latest version of the manuscript. We now provide a more detailed explanation of each parameter that can be adjusted within the platform in our documentation and in the notebooks.

The documentation now reads:
“Apply a moving average technique to the positional data in your tracks. By adjusting the `Smoothing Neighbors` slider, you can control the degree of smoothing. The smoothing of tracks is performed using a moving average technique, which averages the position data over a specified number of neighboring points centered around each data point. This reduces jitter and minor positional fluctuations in the data. For points at the edges where a full window of neighbors isn't available, the original values are used to ensure no data is lost.”

https://github.com/CellMigrationLab/CellTracksColab/wiki/Track-filtering#1-choosing-your-filters

From our understanding, the algorithm calculates speed and persistence of the cell tracks by using the whole length of the track. This represents a limitation, as track speed and persistence are prone to discrepancies based on track length. If one sample has longer tracks than another then this could lead to artificial differences. In the current setup, short and long tracks are grouped together, and their output considered equivalent. An alternative approach would be to run speed and persistence calculations on a pre-defined time window deltaT for each identified track in a moving average manner. The definition of such parameter from the user would be a welcome addition to the notebook.

Thank you for pointing out the limitation in our initial method of calculating track metrics using the entire length of the track. This approach could indeed introduce discrepancies when tracks vary significantly in length, potentially affecting the accuracy of the analysis. In response to your suggestion, we have updated CellTracksColab to include a feature that allows users to define specific time windows for each track. This enhancement enables the calculation of track metrics using a rolling average/sum method. This method is particularly advantageous for metrics not normalized to track length, such as total distance traveled and total turning angles.

https://github.com/CellMigrationLab/CellTracksColab/wiki/Track-Metrics#metrics-computed-using-rolling-windows

Other filtering options would also be useful in specific biological situations, such as filtering out cells that are not moving (short trajectories).
In response to your feedback, we have expanded the filtering options available within the platform. Users can now filter tracks based on several parameters, including duration, mean speed, maximum and minimum speed, and total distance traveled. We are committed to continually enhancing the functionality of CellTracksColab. As such, we plan to introduce additional filtering options as the platform evolves, driven by user feedback and the emerging needs of the scientific community.

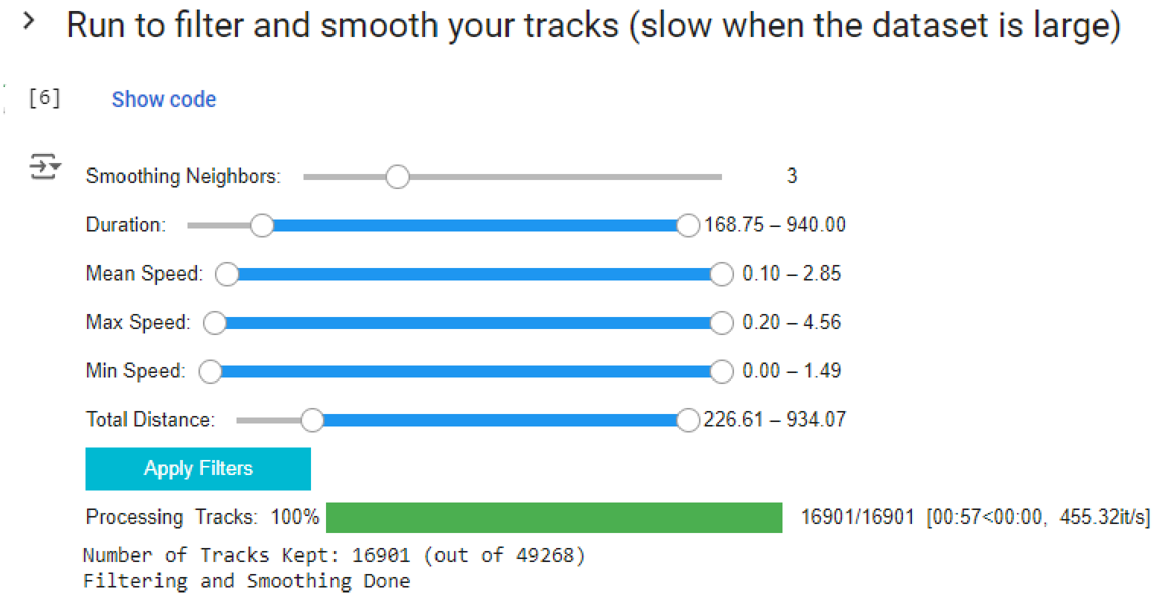

https://github.com/CellMigrationLab/CellTracksColab/wiki/Track-filtering

The notebook is meant to be novice-oriented. While this stands true thanks to the easy installation and the code being "hidden" from view, the tool can become quite overwhelming for novices. One way around would be to separate the current content into different notebooks (one for data visualisation, one for standard analysis, one for quality control and one for advanced analysis and clustering). This would also allow additional space for lengthier explanations where needed (e.g., smoothing, see above), and would prevent user being put off by advanced features they might not be interested with to start with.

Thank you for your insightful feedback. We recognize that as CellTracksColab grows in functionality, it can be challenging to maintain simplicity for novice users while offering advanced features for more experienced users. In response to your suggestions, we have

divided the main notebook into two distinct parts to reduce complexity and improve user navigation:

- This first notebook focuses on the initial stages of the workflow, including data import, computation of track metrics, and basic visualization of track parameters. It's designed to be straightforward and accessible for novices, providing them with the essential tools to start their analysis without being overwhelmed by more complex functionalities.
- The second notebook is tailored for users ready to delve into more sophisticated analyses, such as dimensionality reduction and clustering. This separation allows users to be comfortable with the basic analyses and advance at their own pace without needing to navigate through more advanced features initially.

We hope this approach simplifies the user experience for novices. It also keeps the platform efficient and manageable, especially in environments like Google Colab, where managing multiple notebooks can be cumbersome due to the need to reset the environment with each new notebook.

Moreover, while it's very useful for experienced users to be able to access all the computed metrics, for novices this might be discouraging (Fig 1). One suggestion would be to group them (e.g., spatial metrics: directionality, tortuosity, …; shape metrics: area, perimeter, …) and/or to divide them into basic and advanced measurements, with only the basic ones being displayed to start with. A select/unselect all option would also be a welcome addition.
Figure 1 - a subset of the metrics that can be selected.

In response to the reviewer's suggestion regarding the organization and accessibility of computed metrics for novice and experienced users, we have significantly enhanced the user interface for metric selection. This improvement addresses the needs of all users by grouping metrics into clear, intuitive categories and providing additional functionality for ease of use. We have categorized the metrics into several groups to facilitate a more user-friendly selection process:

Track Metrics: This category includes fundamental metrics such as Track Duration, Mean Speed, Median Speed, Max Speed, Min Speed, Speed Standard Deviation, Total Distance Traveled, Spatial Coverage, Tortuosity, and Total Turning Angle.

Rolling Track Metrics: Metrics in this category are calculated over a rolling window and include Mean Speed Rolling, Median Speed Rolling, Max Speed Rolling, Min Speed Rolling, Speed Standard Deviation Rolling, Total Distance Traveled Rolling, Directionality Rolling, Tortuosity Rolling, Total Turning Angle Rolling, and Spatial Coverage Rolling.

Morphological Metrics: This category encompasses metrics related to shape and size (when available).

Distance to ROI Metrics: These new metrics are calculated relative to regions of interest (ROIs).

In addition to the metrics computed within CellTracksColab, we support metrics computed directly by the tracking software. This ensures users can access all relevant data, whether newly computed or pre-existing, from their tracking software. To make the user interface more intuitive and less overwhelming, metrics are now organized into an expandable and collapsible accordion menu grouped by the categories above. Each category can be individually expanded or collapsed, and all sections are closed by default to prevent overwhelming the user initially. A "Select All" checkbox is provided for each category, allowing users to select or deselect all metrics within a category quickly.

https://github.com/CellMigrationLab/CellTracksColab/wiki/Track-Metrics

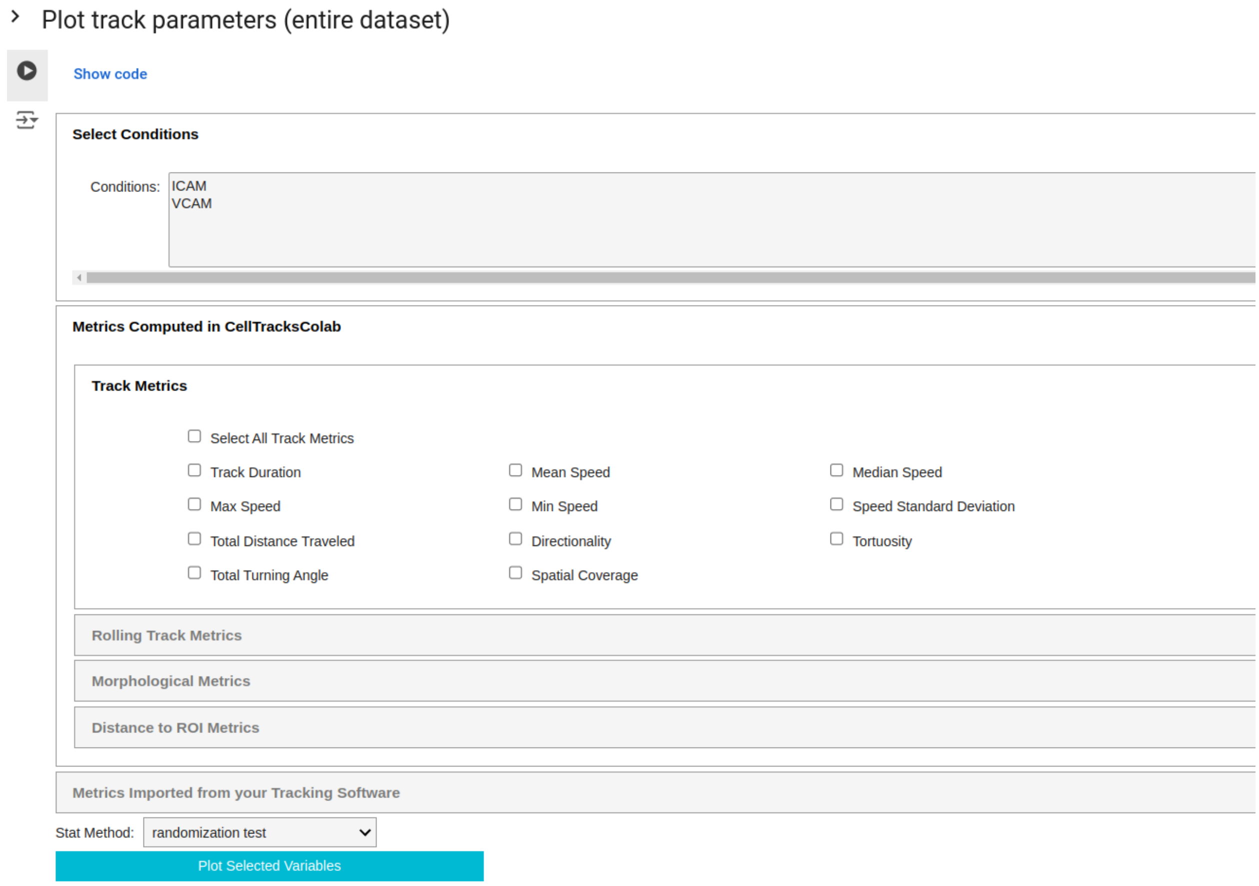

While testing the notebook, a few issues were found, which are listed below.
* In Part 1.3 we could not get to work the "Folder_path" input. We initially downloaded the test dataset separately but could not get the notebook to work when setting either the path to the parent folder, or any of the subfolders in the first input row. Everything works smoothly when choosing "Use_test_dataset".

* Despite setting up the Results folder in the same cell, no output was saved at any point. Confirmation messages of saving were displayed at different point in the notebook (no error messages), but the folder remained empty.

We thank the reviewers for reporting these issues. We have thoroughly tested the platform and hope that most bugs have been fixed.

* In Part 1.5 it is unclear whether the filtering is happening on one movie or on the whole dataset - we assumed it was on the whole dataset, but further clarifications would be helpful. Additional filters would also be welcome (see text above, e.g., filter by distance travelled, deltaT over which analysis should be performed, further details for smoothing)

We thank the reviewer for these suggestions. As described above, we have addressed them.

* In Part 2, it was unclear at first where the generated output is saved. We acknowledge than this might be related to the problem with output saving reported above, but should it not be the case, we think it would be helpful to save the output in csv format for further analysis and perform some additional visualisations at this stage. This would be particularly relevant if splitting the notebook as suggested above.

Thank you for your valuable feedback on the clarity regarding data output in Part 2 of our notebook. To address your concern, we have clarified where and how the generated data is saved in our documentation. In "Part 2: Compute Additional Metrics," all the metrics computed during this phase are saved into a file named merged_Tracks.csv, stored in the designated results folder. This CSV file adheres to a tidy data format, making it highly accessible and easy for further analysis or plotting.

* An error occurred in different sections when trying to plot selected metrics (section 4.1, 4.2.3, 5.7.1, 6.7), despite selecting valid metric calculated in the previous cells:

We thank the reviewers for reporting this issue. We have thoroughly tested the platform and hope that most bugs have been fixed.

* In Part 4.1 the heatmap returns only ±1 values. Is this an expected behaviour? If yes, why would this be the case?

Thank you for bringing this issue to our attention. The observation that the heatmap in Part 4.1 returns only ±1 values arises from an oversight in how z-score normalization was applied. Initially, we applied z-score normalization to the median values calculated for each condition, which could result in extreme values of ±1 in datasets with only two conditions.

This occurred because the z-score transformation exaggerated the difference when only two data points (medians of the two conditions) were used. To address this issue, we have revised the approach to apply z-score normalization across the entire dataset for each track metric before computing the median for each condition. This normalization method considers all data points, providing a more nuanced view of the distribution and reducing the likelihood of extreme values caused by limited data points.

Reviewer #3: Overall, the author provides a useful collection of track analysis tools running on a widely accessible platform. Many different biological use cases generate track data, and so an analysis package has a potentially wide audience. The tools that enable analysis of track heterogeneity will be particularly welcomed by the community.

We thank the reviewer for their supportive comments.

Although the tool itself has many potential users, especially non-programmers, it requires that track data be inputted in a very specific way. The author writes "CellTracksColab processes tracking data stored in CSV files, where each file corresponds to the tracking results from an individual video. These files must be organized and named according to the notebook's specifications for optimal functionality." It's unclear whether a non-programmer could feasibly structure the tracking data in this way.

Thank you for your feedback regarding the accessibility of CellTracksColab for non-programmers, especially concerning the organization and input of tracking data. We understand that requiring data to be formatted and named in a specific way could be a barrier for users unfamiliar with programming or data manipulation.

To address this concern and make the platform more user-friendly:

- CellTracksColab now supports various file types, including CSV and XML, and is designed to be compatible with outputs from multiple popular tracking software platforms (including Trackmate, CellProfiler, Icy, ilastik, and the Fiji Manual Tracker). This compatibility reduces the need for users to manually edit their tracking data files.
- Instead of requiring users to edit file contents, we have simplified the process of organizing files into folders. This structure uses folders to indicate different experimental conditions and repeats.
- We have developed comprehensive documentation and step-by-step guides that illustrate how to organize files correctly. These guides are designed to be easy to follow for non-programmers and include visual aids and examples.
- CellTracksColab includes automated checks that prompt the user if the data appears to be organized incorrectly or if expected files are missing.

These enhancements are designed to make CellTracksColab accessible and manageable for all potential users, including those without programming skills.

A list of software packages that can generate tracks from raw data might also benefit some users.

We thank the reviewer for this excellent suggestion. To facilitate user access to appropriate tools for generating tracking data, we have compiled a list of tracking software packages that are currently compatible with CellTracksColab (Trackmate, CellProfiler, Icy, ilastik, and the Fiji Manual Tracker). This list is readily available on our GitHub webpage.

🔓 Supported Software

CellTracksColab has been tested with tracking outputs from:

TrackMate	CellProfiler	Icy	ilastik	Fiji Manual Tracker

Notes for Specific Software:

- **TrackMate**: Optimized for TrackMate batcher output. Visit TrackMate for more information.
- **CellProfiler**: Compatible with TrackObjects. Visit CellProfiler for more details.
- **Icy**: When using Icy, use the Icy - spot tracking. CSV files must be exported using the 'Track Processor Export to CSV' module.
- **ilastik**: Compatible with ilastik Tracking. Visit ilastik for more details.
- **Fiji Manual Tracker**: Ensure all files have the same column headers. Visit Fiji for more information.
- **Others**: CellTracksColab may also be compatible with other tracking software exporting tracking results that meet our minimal requirements.

https://github.com/CellMigrationLab/CellTracksColab/wiki/Data-requirements-and-supported-software

As argued by the author, the hierarchical clustering does indeed seem to provide clues to the experimenter regarding outliers. Without a sense of statistical significance or robustness, however, it's difficult to use as a scientific measure to compare populations. Are there ways for the user to assess the stability of the clustering?

Thank you for your insightful comment regarding the use of hierarchical clustering in CellTracksColab. While hierarchical clustering can visually suggest the presence of outliers and patterns within the data, relying solely on this technique might not provide the rigorous statistical analysis necessary for robust scientific conclusions. However, this approach is designed to help users identify possible issues with specific fields of view or biological repeats, encouraging experimenters to consider their data differently to ensure accuracy.

Currently, users can assess the robustness of the analysis by varying the clustering and linkage methods to observe differences in the dendrograms produced. We are considering enhancing the clustering module with options like Bootstrap Resampling, which involves repeatedly resampling the dataset with replacement and performing clustering on each sample to assess clusters' stability and robustness against data variations. Although computationally demanding and requiring further validation on multiple datasets to ascertain its utility, this method could significantly deepen the analytical rigor of CellTracksColab. We will explore incorporating these enhancements in future updates.

There seem to be two main rationales for the HDBSCAN clustering of UMAP data, to find actual clusters and to break the data up into categories for fingerprinting. The different rationales aren't clearly described to the reader though, which is especially confusing given that several example datasets don't appear to have natural clusters. Also, similar to the hierarchical clustering, it's not clear how the reader should interpret the fingerprints, especially when more readily interpretable measures are also included in the package. The scatterplots in figures 3 and 4 are too dense to be interpretable. Are there automatic ways that users could avoid generating such plots?

Thank you for the feedback, and we apologize for any confusion caused by our initial presentation of the HDBSCAN clustering of UMAP data in the manuscript. We acknowledge that the dual purposes of using HDBSCAN—for identifying clusters and categorizing data for fingerprinting—were unclear. In response, we have revised the manuscript to clarify these rationales explicitly, ensuring that the objectives and methodologies are transparent and easily understandable for all readers.

Regarding the concerns about the density and interpretability of the scatterplots in Figures 3 and 4, we appreciate your observations. These visualizations are optional and provided as tools for users interested in further exploring UMAP or t-SNE analyses. To aid users in managing the density of these plots, we have introduced features that allow for visualization in both 2D and 3D, along with options to control the spot sizes of individual data points. These improvements are designed to make the visualization tools within CellTracksColab more adaptable and user-friendly, helping all users to produce meaningful and clear graphics that suit their specific analytical needs.

The machine learning for each dataset employs many track metrics that are listed, but not mathematically defined. Where are these metrics defined?

We appreciate the reviewer's observation regarding the need for mathematical definitions for the track metrics employed in our machine-learning analyses. CellTracksColab utilizes a hybrid approach that collects pre-generated track metrics from the tracking software and computes additional metrics based on the track coordinates. This approach allows for a comprehensive analysis but introduces variability in the metrics depending on the tracking software used. Acknowledging the potential confusion this may cause, we have taken significant steps to improve our documentation. The updated documentation now includes detailed descriptions of each track metric that CellTracksColab may display when loading tracks from supported software, whether pre-generated by tracking software or calculated within our platform. Additionally, we guide users in determining the origin of each metric, ensuring clarity and helping users understand and effectively utilize their data within our platform.

https://github.com/CellMigrationLab/CellTracksColab/wiki/Track-Metrics

---

Reviewer #4: Overall impression:
The author presents the tool 'CellTracksColab' for analyzing and visualizing tracking data and also performing downstream statistical analyses and visualization. The described tool appears to be multifunctional and promising and the manuscript is well-written and well-illustrated.

Specific points gathered from the manuscript and while trying out the tool hands-on that speak to the strengths of the tool:
-The authors mention that more than 50,000 tracks, amounting to a staggering ~3 million objects were analyzed using CellTracksColab
-The tool enables automated tracking from videos
-The tool leverages the strength of Python programming language and runs on Google Colab, and thereby makes tracking analysis more accessible for uses with limited programming experience
-Quality control and version control steps are built into the analysis workflow
-While performing track filtering and smoothing, the tool allows side-by-side visualization of 'before' and 'after' graphs, which is helpful
-The graphs present in the manuscript can be generated (and thereby checked) by running the test dataset

We thank the reviewer for taking the time to evaluate our work and providing supporting feedback.

Potential areas of improvement:
-Some sections of the notebooks (mentioned below) could be improved / could be better

documented to help novice image analysts. Video tutorials could also be helpful in this regard.

We thank the reviewers for this comment. We have improved the documentation throughout the notebooks and provided video tutorials.

Video tutorials are available here:
https://github.com/CellMigrationLab/CellTracksColab/wiki

-It is a bit hard to understand how the tool is performing quality control

Thank you for your feedback regarding the clarity of quality control processes within CellTracksColab. We realize the importance of making these processes transparent.

- Quality control within CellTracksColab is implemented at multiple levels to ensure rigorous analysis:
- The tool initially performs automated checks to ensure that all datasets meet the necessary requirements, including the presence of expected files and formats.
- The platform automatically checks for errors within the data, such as inconsistencies in tracking calibration across files, which could affect the analysis's accuracy.
- Users can employ hierarchical clustering to visually assess the presence of outliers and patterns within the data. This feature helps identify anomalies related to specific fields of view or biological repeats, thus prompting a reconsideration of particular datasets.
- The tool allows users to visualize biological repeats individually within the boxplot, aiding in identifying and understanding any discrepancies or anomalies at the biological repeat level.
- Users can use resampling methods to standardize the number of tracks used in each analysis to ensure consistency in comparisons across biological repeats.

We have updated and expanded the documentation to describe these quality control steps.

-I was not successful in running the analysis by choosing 'raw data' and not the 'filtered and smoothed data' for the test dataset using the CellTracksColab TrackMate notebook.

We thank the reviewers for reporting this issue. We have thoroughly tested the platform and hope that most bugs have been fixed.

-While trying to plot the balanced dataset, the error message 'no parameters are selected' is often received, even when parameters have been set.

We thank the reviewers for reporting this issue. We have thoroughly tested the platform and hope that most bugs have been fixed.

-Runtime speeds provided by Google Colab (as the author also mentions in the manuscript) can be a challenge

We acknowledge the concerns regarding the runtime speeds experienced on Google Colab, as mentioned in the manuscript. To address this, we have introduced the option to run CellTracksColab locally, which can significantly alleviate speed issues, especially for users with larger datasets. Running the platform locally leverages the user's computing resources, thus bypassing the limitations sometimes faced on cloud platforms like Google Colab. In addition to providing a local running option, we have optimized the code by identifying and improving several bottlenecks within our processing workflows. These optimizations enhance the overall processing speed by using multithreading, making the platform more efficient regardless of whether it's run locally or in the cloud.

https://github.com/CellMigrationLab/CellTracksColab/wiki/Running-CellTracksColab-locally

-The author recommends arranging data into a specific folder hierarchy comprising conditions and repeats. For users with a smaller dataset (e.g. without multiple conditions and/or repeats), it might be helpful to not make this hierarchy mandatory for using the tool or supporting a flat architecture instead.

We appreciate the reviewer's thoughtful suggestion regarding data organization within CellTracksColab. We recognize that our initial guidance on arranging data into a specific folder hierarchy may be less applicable for users with smaller datasets, such as those containing only one condition or repeat.

To clarify, our platform does not require a minimum number of conditions or repeats to function. For users with smaller datasets, organizing data can indeed be straightforward. The simplest structure necessary follows a single condition and a single repeat, organized as Condition > Repeat 1 > Tracks. This structure allows CellTracksColab to process the data efficiently while maintaining the organizational benefits that our folder hierarchy provides.

We have updated our documentation to explicitly include instructions for users with smaller datasets, ensuring they understand how to structure their data appropriately.
