# Supplementary material for: CellTracksColab is a platform that enables compilation, analysis, and exploration of cell tracking data
Source: PLoS Biol. 2024 Aug 8;22(8):e3002740. doi: 10.1371/journal.pbio.3002740 (PMC11335138; doi:10.1371/journal.pbio.3002740)
Supplement: Attachment — Submitted filename: Rebuttal.pdf [file pbio.3002740.s009.pdf]

Reviewer #1: The authors have addressed all of my comments in full. I'd like to thank them for their efforts to tackle the issues I raised and I think the platform and paper are much better as a result.

We thank the reviewer for supporting the publication of our manuscript.

Reviewer #3: The authors addressed all of my comments well. They especially now provide multiple documented ways for users to input tracking data, which will expand their audience. They created software that many people will hopefully soon use.

We thank the reviewer for supporting the publication of our manuscript.
