# Supplementary material for: CellTracksColab is a platform that enables compilation, analysis, and exploration of cell tracking data
Source: PLoS Biol. 2024 Aug 8;22(8):e3002740. doi: 10.1371/journal.pbio.3002740 (PMC11335138; doi:10.1371/journal.pbio.3002740)
Supplement: Attachment — Submitted filename: CellTracksColab review.docx [file pbio.3002740.s007.docx]

The authors of CellTracksColab present an interesting platform for analysing image tracking data, which appears very useful for the community. However, some points should be addressed to make the suggested tool more accessible.

One question is regarding how some of their metrics are calculated within the platform and which parameters can be tuned. For example, the smoothing option is described in the notebook as “Smoothing: Adjust this slider to control the degree of smoothing you'd like to apply to your tracks.” – what is the slider defining? The temporal interval of a moving average? It is possible to retrieve this information by expanding the code cells in the notebook, but this is only true for experienced (coding) users.

From our understanding, the algorithm calculates speed and persistence of the cell tracks by using the whole length of the track. This represents a limitation, as track speed and persistence are prone to discrepancies based on track length. If one sample has longer tracks than another then this could lead to artificial differences. In the current setup, short and long tracks are grouped together, and their output considered equivalent. An alternative approach would be to run speed and persistence calculations on a pre-defined time window deltaT for each identified track in a moving average manner. The definition of such parameter from the user would be a welcome addition to the notebook.

Other filtering options would also be useful in specific biological situations, such as filtering out cells that are not moving (short trajectories).

The notebook is meant to be novice-oriented. While this stands true thanks to the easy installation and the code being “hidden” from view, the tool can become quite overwhelming for novices. One way around would be to separate the current content into different notebooks (one for data visualisation, one for standard analysis, one for quality control and one for advanced analysis and clustering). This would also allow additional space for lengthier explanations where needed (e.g., smoothing, see above), and would prevent user being put off by advanced features they might not be interested with to start with.

Moreover, while it’s very useful for experienced users to be able to access all the computed metrics, for novices this might be discouraging (Fig 1). One suggestion would be to group them (e.g., spatial metrics: directionality, tortuosity, …; shape metrics: area, perimeter, …) and/or to divide them into basic and advanced measurements, with only the basic ones being displayed to start with. A select/unselect all option would also be a welcome addition.

Figure 1 - a subset of the metrics that can be selected.

While testing the notebook, a few issues were found, which are listed below.

- In Part 1.3 we could not get to work the “Folder_path” input. We initially downloaded the test dataset separately but could not get the notebook to work when setting either the path to the parent folder, or any of the subfolders in the first input row. Everything works smoothly when choosing “Use_test_dataset”.

- Despite setting up the Results folder in the same cell, no output was saved at any point. Confirmation messages of saving were displayed at different point in the notebook (no error messages), but the folder remained empty.

- In Part 1.5 it is unclear whether the filtering is happening on one movie or on the whole dataset – we assumed it was on the whole dataset, but further clarifications would be helpful. Additional filters would also be welcome (see text above, e.g., filter by distance travelled, deltaT over which analysis should be performed, further details for smoothing)
- In Part 2, it was unclear at first where the generated output is saved. We acknowledge than this might be related to the problem with output saving reported above, but should it not be the case, we think it would be helpful to save the output in csv format for further analysis and perform some additional visualisations at this stage. This would be particularly relevant if splitting the notebook as suggested above.
- An error occurred in different sections when trying to plot selected metrics (section 4.1, 4.2.3, 5.7.1, 6.7), despite selecting valid metric calculated in the previous cells:

- In Part 4.1 the heatmap returns only ±1 values. Is this an expected behaviour? If yes, why would this be the case?
